# Improving outcomes in co-morbid diabetes and COVID-19: A quasi-experimental study

**DOI:** 10.4102/safp.v65i1.5631

**Published:** 2023-02-13

**Authors:** Tatum Aronson, Joel Dave, Tasleem Ras

**Affiliations:** 1 Division of Family Medicine, Department of Family, Community and Emergency Care, Faculty of Health Sciences, University of Cape Town, Cape Town, South Africa; 2 Division of Endocrinology, Department of Medicine, Faculty of Health Sciences, University of Cape Town, Cape Town, South Africa

**Keywords:** COVID-19, diabetes mellitus, health systems, primary health care, intermediate care, family medicine, clinical risk classification

## Abstract

**Background:**

High-risk people living with diabetes (PLWD) have increased risk for morbidity and mortality. During the first coronavirus disease 2019 (COVID-19) wave in 2020 in Cape Town, South Africa, high-risk PLWD with COVID-19 were fast-tracked into a field hospital and managed aggressively. This study evaluated the effects of this intervention by assessing the impact of this intervention on clinical outcomes in this cohort.

**Methods:**

A retrospective quasi-experimental study design compared patients admitted pre- and post-intervention.

**Results:**

A total of 183 participants were enrolled, with the two groups having similar demographic and clinical pre-Covid-19 baselines. Glucose control on admission was better in the experimental group (8.1% vs 9.3% [*p* = 0.013]). The experimental group needed less oxygen (*p* < 0.001), fewer antibiotics (*p* < 0.001) and fewer steroids (*p* = 0.003), while the control group had a higher incidence of acute kidney injury during admission (*p* = 0.046). The median glucose control was better in the experimental group (8.3 vs 10.0; *p* = 0.006). The two groups had similar clinical outcomes for discharge home (94% vs 89%), escalation in care (2% vs 3%) and inpatient death (4% vs 8%).

**Conclusion:**

This study demonstrated that a risk-based approach to high-risk PLWD with COVID-19 may yield good clinical outcomes while making financial savings and preventing emotional distress.

**Contribution:**

We propose a risk-based approach to guide clinical management of high risk patients, which departs significantly from the current disease-based model. More research using randomised control trial methodology should explore this hypothesis.

## Introduction

In November 2019, the world was informed of the first cases of coronavirus disease 2019 (COVID-19), caused by severe acute respiratory syndrome coronavirus 2 (SARS-CoV-2), which rapidly escalated into a full-blown pandemic in early 2020. In Italy, it was noted that diabetes mellitus (DM) was three times more prevalent in patients with severe COVID-19 than in the general population.^1^ It was thus anticipated that DM would also predispose people to increased severity of COVID-19 in South Africa.^1^

South Africa is a lower-middle-income country that lacks data on the role of intermediate care services in the health system.^2^ In preparation for the COVID-19 pandemic, the government set up field hospitals to ensure that patients infected with COVID-19 would be adequately treated in an already strained health system. In Cape Town, the first field hospital, the 862-bed Hospital of Hope (HoH), was erected in the Cape Town International Convention Centre (CTICC). It was set up in May 2020 and admitted the first patient on 08 June 2020. The hospital offered inpatient intermediate care which included oxygen support, intravenous fluid and medical management, access to mobile x-rays, physiotherapists, dieticians, social workers, an onsite pharmacy and support from a nearby laboratory.^3^

Diabetes mellitus is a global public health problem and is one of the leading causes of morbidity and mortality worldwide.^1^ This is especially concerning in South Africa, where the healthcare system is not only overwhelmed by the escalating prevalence of noncommunicable diseases (NCDs) but carries an additional burden of disease as a result of the tuberculosis and human immunodeficiency virus (HIV) epidemics.^1^

Diabetes mellitus can be regarded as a chronic inflammatory condition characterised by multiple metabolic and vascular abnormalities. There is also a dysregulated immune response increasing the diabetic patient’s risk of infections.^4^ This, together with an augmented inflammatory process, may contribute to the underlying mechanism that leads to a higher propensity to infections with worse outcomes.^4^ Evidence suggests that SARS-CoV-2 induces a vigorous innate immune response, leading to a ‘cytokine storm’ which is thought to play a critical role in the high mortality of patients with COVID-19.^5^ The ‘cytokine storm’ is a crucial cause of acute respiratory distress syndrome (ARDS), a systemic inflammatory response, and multiple organ failure.^6^ The onset of dyspnoea and ARDS usually occurs at a median of 5 and 8 days, respectively.^7^ Recently, the pulmonary pathology of SARS-CoV-2 infection was shown to be diffuse alveolar damage, alveolar oedema with proteinaceous exudates, thickening of alveolar walls, desquamation of pneumocytes and hyaline membrane formation, all indicative of ARDS.^8^ The presence of Type 2 DM (T2DM) with chronic inflammation and other associated comorbidities may allow unrestricted viral replication and trigger heightened levels of inflammation and hyperimmune reaction, greatly exacerbating the response to SARS-CoV-2.^5^

A retrospective multicentre study in Hubei province, China, investigated 952 patients with pre-existing T2DM who were also diagnosed with COVID-19. This study suggested that people living with diabetes (PLWD) required more medical interventions and had a significantly higher mortality (7.8% vs 2.7%; adjusted hazard ratio [aHR], 1.49) and multiple organ injuries than those without DM.^9^ This study also found that if glycaemic variability was maintained between 3.9 mmol/L and 10 mmol/L, there was a significant reduction in medical interventions, major organ injuries and all-cause mortality.^9^ Glycaemic variability has been shown to be an important indicator and a possible risk predictor for death and other complications in individuals with T2DM.^10^ Efforts to ensure good inpatient glycaemic control are therefore the cornerstone in the management of PLWD who are diagnosed with COVID-19. To obtain good glycaemic control in the context of field hospitals with rapid turnover of patients and inexperienced staff, the use of clinical protocols assumes increased importance.

In a systematic review and meta-analysis that included 475 publications, the weighted prevalence of mortality in hospitalised COVID-19 patients with DM (20.0%, 95% confidence interval [CI]: 15.0–26.0; *I*^2^, 96.8%) was 82% (1.82 times) higher compared to that in non-DM patients (11.0%, 95% CI: 5.0–16.0; *I*^2^, 99.3%). The prevalence of mortality among DM patients was highest in Europe (28.0%; 95% CI: 14.0–44.0) followed by the United States (20.0%, 95% CI: 11.0–32.0) and Asia (17.0%, 95% CI: 8.0–28.0). The weighted prevalence of DM among hospitalised COVID-19 patients was 20% (95% CI: 15–25, I2, 99.3%).^23^

National guidelines and standards of care for DM are now available in many countries.^11^ Translation of practice recommendations from developed countries to the practical care of PLWD living in developing countries is challenging as there is differential access to various aspects of care.^11^

Data from the Western Cape Department of Health showed that COVID-19 and DM comorbidity had dramatically higher mortality rates than COVID-19 patients without DM.^12^ Local data also similarly demonstrated that there is an increased mortality in patients diagnosed with COVID-19 who were older in age and had DM, hypertension and renal impairment.^13^ It was therefore decided to offer high-risk PLWD an elective admission to the CTICC HoH, with the hypothesis that this would prevent increased morbidity and mortality. Because there was a lack of robust scientific data, a consensus document on inpatient DM management in the form of inpatient practice guidelines was adapted from a nearby tertiary hospital and implemented at the HoH (named the high-risk diabetes–COVID-19 protocol – HRDCp – Appendix 1).

The aim of this study was to assess the impact of early elective admission of high-risk PLWD diagnosed with COVID-19 and the application of a clinical practice guideline (HRDCp) on clinical outcomes in a generalist-run intermediate care facility. The following objectives were fulfilled: a description of the demographics and baseline characteristics of participants; a description of the inpatient clinical course of this cohort, comparing the control and experimental groups; a description of the clinical outcomes of this cohort, comparing the control and experimental groups.

## Research methods and design

### Study setting

The district health system in the Western Cape comprises six districts, five rural and one located in urban Cape Town. The Cape Town Metro district is further subdivided into eight subdistricts paired to form four substructures. It is to the substructure level that governance powers are decentralised. Hospitals in all these Metro substructures, and tertiary hospitals in Cape Town, referred patients to the HoH according to agreed-upon referral criteria (Appendix 2).

The HoH medical staff comprised seven teams. Each team had a team leader, who was a senior clinician in family medicine (five teams), internal medicine (one team) or emergency medicine (one team). The HRDCp was implemented in the treatment of the experimental cohort of patients for inpatient management. Patients were preferably admitted for at least 8 days to ensure that they did not decompensate during the time period in which the ‘cytokine storm’ was expected to occur.

### Study design

This was a retrospective quasi-experimental study. This study design was used to assess a real-world intervention, retrospectively, between two predefined groups of PLWD.

### Study population

High-risk PLWD satisfying the inclusion criteria were identified by using a database from the Western Cape Data Centre. Code-named VECTOR (Virtual Emergency Care Tactical OpeRation), a group of medical officers obtained data from the data centre, which ran an algorithm to generate a list of high-risk PLWD with a COVID-19 diagnosis in a 10-day window.^13^ These patients were then allocated to the medical officers who contacted them telephonically to offer them elective admission to the HoH.

All 61 patients who accepted admission to the HoH via the telemedicine (VECTOR) community group were included as the experimental group, while 122 purposively selected patients matching the inclusion criteria below in a 2:1 ratio were identified from those admitted prior to the introduction of the intervention (HRDCp) to make up the control group. The two groups were matched for age, gender and renal function.

Inclusion criteria were Type I or II diabetes mellitus with COVID-19 (polymerase chain reaction [PCR] test or clinical diagnosis) *and* renal impairment (creatinine of more than 100) *or* age older than or equal to 65 years of age.

Exclusion criteria were age younger than 65 and normal renal function, and for controls, the exclusion criteria were being admitted after the HRDCp was introduced and not being part of the VECTOR cohort.

Renal impairment was defined using the RIFLE (risk, injury, failure, loss of kidney function and end-stage kidney disease) criteria, which included a rise in creatinine, which results in risk, injury, failure, loss of kidney function and end-stage kidney disease (see Appendix 3).^24^

### Data collection

Data were extracted from the HoH clinical database (described earlier) using a data extraction tool that was designed by the research team, piloted on five PLWD who were not part of this study and modified accordingly. The study period covered the entire duration of the facility’s operation, from June 2020 to August 2020.

The data extraction tool is attached as Appendix 4.

### Statistical analysis

Data were analysed using Stata 14 (StataCorp LLC, College Station, Texas, United States). Patient characteristics, comorbidities and outcomes were compared using χ^2^ or Fisher’s exact tests for categorical data and Wilcoxon rank-sum (Mann–Whitney) tests for continuous data. All statistical tests were two-sided with significance set at α = 0.05.

## Results

Figure 1 shows the care pathways of participants in the study. Sixty-one patients accepted the offer for elective admission via the telemedicine group, forming the experimental group. For the control group, 122 PLWD were identified from the dataset and included in this group. The baseline characteristics and demographics of both populations are shown in Table 1.

**FIGURE 1:**
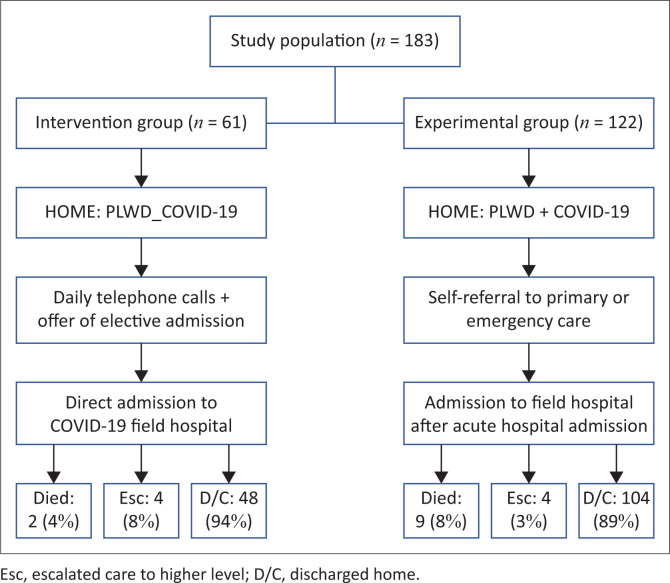
Care pathways for intervention and control cohorts. Missing data for final outcome (*n* = 15 participants).

**TABLE 1:** Baseline characteristics of the study population.

Variable	Total sample				Experimental group				Control group				*p*	Missing data (*n*)
	Median	IQR	*n*	%	Median	IQR	*n*	%	Median	IQR	*n*	%		
Number of patients	183				61				122				-	-
**Age**	66	62–71	-	-	66	65–71	-	-	65	65–71	-	-	-	-
**Gender**														
Female	-	-	111	61	-	-	37	61	-	-	74	61	-	-
Male	-	-	72	39	-	-	24	39	-	-	48	39	-	-
HbA1c	9.1	7.2–11.1	-	-	8.1	7.1–10.3	-	-	9.3	7.3–12.1	-	-	0.013	7
Creatinine	81	66–104	-	-	78	65–100	-	-	83	66–110	-	-	-	1
eGFR	65	52–91	-	-	61	52–90	-	-	66	52–92	-	-	0.548	2
Hb	12.8	11.5–14.1	-	-	12.4	11.4–13.9	-	-	12.8	11.6–14.1	-	-	0.406	11
HGT at admission	10.6	7.2–14.5	-	-	10.2	6.6–13.1	-	-	10.7	7.8–15.3	-	-	0.039	39
**Comorbidities**														
Hypertension	-	-	141	82	-	-	44	85	-	-	97	82	0.624	-
Chronic obstructive pulmonary disease (COPD)	-	-	21	12	-	-	3	6	-	-	18	15	0.127	-
Smoking	-	-	28	16	-	-	6	12	-	-	22	18	0.259	-
Overweight or obesity	-	-	10	6	-	-	5	10	-	-	5	4	0.174	-
Ischaemic heart disease	-	-	25	15	-	-	10	19	-	-	15	13	0.259	-
Chronic kidney disease	-	-	8	5	-	-	1	2	-	-	7	6	0.437	-
HIV	-	-	17	10	-	-	2	4	-	-	15	13	0.098	-
Congestive cardiac failure	-	-	80	47	-	-	23	44	-	-	57	48	0.658	-

Note:Individual patients may have more than one comorbidity. Missing data: data not available from clinical records or database.

eGFR, estimated glomerular filtration rate; HGT, haemoglucotest; HIV, human immunodeficiency virus; IQR, interquartile range.

The two groups were similarly matched for age (median 66 years vs 65 years), gender (identical ratios) and renal function (estimated glomerular filtration rate [eGFR] [mL/min/1.73 m^2^] 61 vs 66; *p* = 0.546).

There was no significant difference in any comorbidity between the groups (Table 1), with the data indicating hypertension, chronic kidney disease, overweight or obesity and cardiac disease to be prevalent in both groups.

On admission, the experimental group had significantly better diabetes control than the control group (HbA1c 8.1% [7.1, 10.3] vs 9.3% [7.3, 12.1], *p* = 0.013); admission random glucose 10.2 mmol/L (6.6, 13.1) versus 10.7 mmol/L (7.8, 15.3), *p* = 0.039.

Table 2 shows the clinical interventions that were administered during admission. The majority of participants in the experimental group (73%) only required room air in contrast to the participants in the control group, where the majority required oxygen. Fifty-six percent (11) required nasal cannula oxygen, and 28% required some type of face mask oxygen. Participants in the experimental group required non-rebreather facemasks as their highest level of oxygen (2%) versus 12% in the control group (*p* = 0.041), while some matched controls additionally required double-barrel oxygen (3%) as their highest level of oxygen requirement.

**TABLE 2:** Inpatient glycaemic control and interventions.

Variable	Total sample				Experimental group				Control group				*p*	Missing data (*n*)
	Median	IQR	Absolute no.	Proportion (%)	Median	IQR	Absolute no.	Proportion (%)	Median	IQR	Absolute no.	Proportion (%)		
HGT	9.4	7.6–11.8	-	-	8.3	6.9–10.4	-	-	10.0	7.7–12.5	-	-	0.006	39
One or more hypoglycaemic episodes during admission	-	-	44	27	-	-	13	25	-	-	31	28	0.721	22
One or more hyperglycaemic episodes during admission	-	-	117	82	-	-	37	82	-	-	80	82	0.932	40
HGT at discharge	7.7	6.3–10.2	-	-	7.4	5.3–9.4	-	-	8.1	6.6–10.8	-	-	0.032	32
Insulin at admission	-	-	63	37	-	-	18	36	-	-	45	38	0.824	14
Insulin at discharge	-	-	85	51	-	-	22	44	-	-	63	54	0.244	16
Dietician consult	-	-	54	37	-	-	21	46	-	-	33	33	0.141	37
**Oxygen**														
Room air	-	-	60	35	-	-	38	73	-	-	22	18	< 0.001	11
Nasal cannula	-	-	78	45	-	-	11	21	-	-	67	56	< 0.001	11
Face mask (40%)	-	-	17	10	-	-	2	4	-	-	15	13	0.071	18
Non-rebreather mask	-	-	15	9	-	-	1	2	-	-	14	12	0.041	11
Double barrel†	-	-	3	2	-	-	0	-	-	-	3	3	0.554	11
Days on oxygen	2	0–5	-	-	0	0–1	-	-	4	1–6	-	-	< 0.001	11
Antibiotic use	-	-	46	27	-	-	5	9	-	-	41	35	< 0.001	13
**Steroid use**	-	-	80	47	-	-	16	30	-	-	64	55	0.003	13
Oral use	-	-	18	11	-	-	0	0	-	-	-	-	-	-
Intravenous use	-	-	-	-	-	-	-	-	-	-	18	15	0.001	-

Note: Missing data: data not available from clinical records or database.

HGT, haemoglucotest; IQR, interquartile range.

† , Non-rebreather face mask oxygen plus nasal cannula oxygen.

Antibiotics were more commonly used in the control group (35% vs 9%; *p* < 0.001). Corticosteroids (oral and intravenous) were also more commonly used in the control group (55% vs 15%; *p* < 0.005).

Additionally, participants in the experimental group had significantly lower admission finger-prick glucose results compared to those in the control group (8.3 mmol/L vs 10.0 mmol/L, *p* = 0.006). The discharge glucose levels, insulin requirements and glucose-related adverse events were similar in both groups.

Table 3 indicates the adverse events and clinical outcomes that occurred in the two groups. Participants in the experimental group had a shorter hospital stay than those in the control group (5 days vs 6 days, *p* = 0.04). Acute kidney injury (AKI) occurred less frequently in the experimental group (8% vs 20%, *p* = 0.046). Fewer participants in the experimental group required escalation of care (2% vs 8%, *p* = 0.286), although this was not statistically significant. Similarly, a lower proportion of participants in the experimental group died in hospital, although the difference was not statistically significant (4% vs 8%; *p* = 0.508). The proportion of patients discharged home was similar (94% vs 89%).

**TABLE 3:** Adverse events and clinical outcomes of study participants.

Variable	Total sample				Experimental group				Control group				*p*	Missing data (*n*)
	Median	IQR	*n*	%	Median	IQR	*n*	%	Median	IQR	*n*	%		
Number of patients	183				61				122				-	-
Median duration of stay: days	6	3–8	-	-	5	3–8	-	-	6	4–9	-	-	0.040	12
Adverse events†	-	-	28	16	-	-	4	8	-	-	24	20	0.046	11
AKI DKA	-	-	5	3	-	-	0	0	-	-	5	4	0.324	-
Acute confusional state	-	-	8	5	-	-	0	0	-	-	8	7	0.108	-
Hypoglycaemia	-	-	4	2	-	-	1	2	-	-	3	3	1.000	-
Other	-	-	8	5	-	-	2	4	-	-	6	5	1.000	-
Escalation to acute care	-	-	10	6	-	-	1	2	-	-	9	8	0.286	11
Mortality	-	-	11	6	-	-	2	4	-	-	9	8	0.508	11
Disposition or	-	-	152	90	-	-	48	94	-	-	104	89	0.667	15
discharge home	-	-	5	3	-	-	1	2	-	-	4	3	-	-
Acute care	-	-	11	7	-	-	2	4	-	-	9	8	-	-
Death	-	-	-	-	-	-	-	-	-	-	-	-	-	-

Note: Missing data: data not available from clinical records or database.

IQR, interquartile range; AKI, acute kidney injury; DKA, diabetic ketoacidosis.

† , Individual patients may appear in more than one subgroup.

The experimental group had significantly better diabetes control than the control group (HbA1c 8.1% [7.1, 10.3] vs 9.3% [7.3, 12.1], *p* = 0.013). The discharge glucose levels, insulin requirements and glucose-related adverse events were similar in both groups.

In the experimental group, there was a shorter hospital stay (5 days vs 6 days, *p* = 0.04), less occurrence of AKI (*p* = 0.046), less escalation of care (*p* = 0.286) and less mortality (*p* = 0.508), although the latter two indicators were not statistically significant.

## Discussion

The key findings of this study relate to the clinical course and outcomes in two groups of PLWD. High-risk PLWD in the experimental group were managed with good outcomes at a field hospital. This will improve confidence in the future for the down-referral of high-risk PLWD to intermediate care facilities. These clinical outcomes were achieved without needing admission to an acute hospital first, implying savings in cost and possibly improved patient experience, although these were not measured in this study.

Although the control and experimental cohorts were similar at pre-COVID-19 status, their COVID-19 clinical parameters were markedly different, possibly explaining the significant differences in oxygen need, steroid use, antibiotic administration and glycaemic control. What is known in this area is that hyperglycaemia, increased coagulation rate and elevated release of pro-inflammatory cytokines all facilitate the severity of COVID-19 in PLWD.^14^ The authors suspect that the intervention of early elective admission, followed by tight glycaemic control, may have prevented the cytokine storm in this cohort.

Further studies in this regard would be useful to evaluate and prove this hypothesis.

Acute kidney injury was found to be significantly more prevalent in the control group; this is likely to be multifactorial but ultimately highlights that these participants had more severe COVID-19. In a study performed in Turkey which included 578 patients, the incidence of AKI at admission was higher in patients with chronic kidney disease (CKD) than those without CKD (52.1% vs 39.3% respectively, *p* = 0.006).^15^ In a study of 4020 consecutively hospitalised patients in Wuhan, China, 285 were identified as having AKI and had an increased risk of inpatient mortality.^16^ Acute kidney injury, regardless of the cause, remains a key adverse event that clinicians should be wary of.

This study demonstrated that the most common comorbidity among PLWD with COVID-19 at the HoH was hypertension. This is similar to other studies. In New York, a study among 5700 hospitalised patients with COVID-19 showed that 1808 patients (33.8%) had diabetes and 3026 (56.6%) had systemic hypertension, while 1737 (41.7%) were obese.^17^ Although the body mass index (BMI) was recorded as raised in the current study where it was measured, exact values were not recorded on all patients, and this was therefore difficult to interpret accurately. However, the link between obesity and diabetes is clear, and this remains an important clinical risk to be observed in this population.^18^

Using a tertiary-level clinical guideline proved to be a feasible option in this study, resulting in safe and effective management of diabetes compared to usual care. In the context of diabetes–COVID-19 in a field hospital, where tight glycaemic control is an important goal, realistic protocols are important tools to guide management decisions. A multicentred study in Hubei of over 7000 cases of COVID-19 reported a significant correlation between well-controlled blood glucose and lower levels of inflammatory markers.^9^ In Michigan, tailored protocols and algorithms were developed to improve glycaemic control for 200 patients admitted with COVID-19, and this allowed them to react to surges in glucose levels driven by disease activity and reduce the burden on the primary teams.^19^

An important finding is that the clinical outcomes of the two groups were similar. Based on their pre-COVID-19 morbidity profile, participants in the experimental group represented a group that had a high likelihood of requiring admission, needing critical care and dying.^12,13^ In this group, 94% were discharged home without having to potentially endure the anxieties associated with admission to an acute hospital, most often via an emergency centre. While the authors did not include anxiety or depression measurements in this study, several Chinese studies make the link between anxiety disorders and acute hospital admission for COVID-19.^20,21,22^ These findings suggest a significant missed opportunity in this context for learning about the impact of acute hospital admission on the mental health of PLWD, and this should inform future research.

Limitations of the study include the small sample size, missing clinical data and the fact that this study compared PLWD with differing levels of severity of COVID-19. A further limitation is that this study did not include longer-term follow-up data. The quasi-experimental design has implicit limitations in that it does not use random sampling in constructing experimental and control groups and has low internal validity. Using nonuniform comparison groups can limit generalisation of the findings, because noncontrolled variables may have influenced the results. While useful for health systems research, the strength of the evidence is not equal to a randomised controlled trial because of the uncontrolled confounding factors in real life.

## Conclusion

This study compared a novel approach to managing risk for adverse outcomes by early admission and tight diabetes control to the usual practice of waiting for severe disease to arise and subsequent emergency admission. While showing similarity to usual care in terms of clinical outcomes in the context of a field hospital, it is suggested that savings were made in terms of medical complications and acute admissions costs (financial and emotional).

Further studies should look at how digital innovations could enhance the coordination of care across all levels of the health system, the role of clinical risk factors as criteria for elective escalation of healthcare and ways to enhance interdisciplinary, interfacility and vertical collaborations. Specifically, attention should be paid to the cost-effectiveness of novel interventions and the psychosocial impact of these interventions.

## Figures and Tables

**FIGURE 1-A1:**
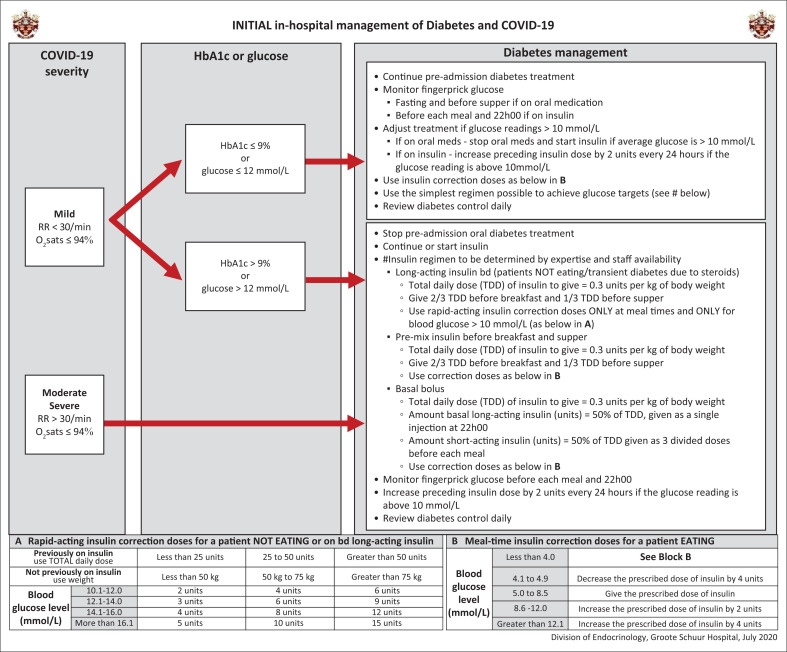
Initial in-hospital management of diabetes and COVID-19.

**FIGURE 1-A4:**
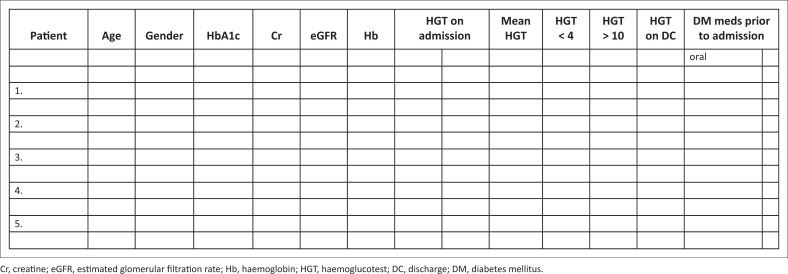
Data extraction tool for admission and in-patient clinical variables.

**FIGURE 2-A4:**
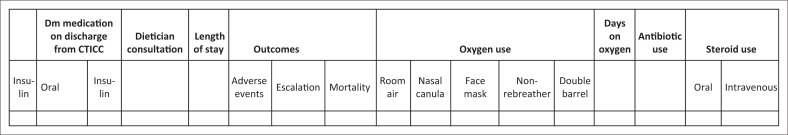
Data extraction tool for discharge summary.

## Appendix 2

**TABLE 1-A2:** Intermediate care bed facility (ICBF) admission criteria.

Category	Characteristics and admission criteria
I – COVID-19 only	Mobile and independent Younger adults or adolescents (> 16 years) deemed mature enough to give informed consent Needs oxygen support Normal level of consciousness Minimal or no comorbidities – key medical problem is COVID-19 pneumonia Pregnant women in the first trimester of an uncomplicated pregnancy
II – COVID-19 + comorbid	Variable mobility and independence Has comorbidities, that is, clinical complexity, including: diabetes, HIV, obesity, hypertension – may outweigh the COVID-19 issues in clinical acuity May need oxygen – if comorbid condition has stabilised and needs ongoing in-patient care, and not suitable for quarantine and isolation facility
III – COVID-19 + palliative	Severely limited mobility and ADL’s Palliative care, that is, symptom control and optimised quality of life Palliation may be due to COVID-19 or non-COVID-19 May need oxygen, or cannot go home or isolation facility

HIV, human immunodeficiency virus; ADL, activities of daily living; COVID-19, coronavirus disease 2019.

Note: The ICBFs will accept COVID-19 patients regardless of the availability of a laboratory diagnostic confirmation – the clinical diagnosis of COVID-19 by the referring clinician is deemed sufficient.

**Not suitable for admission:**

- need for ventilation
- need for inotropes
- need for significant levels of monitoring, for example, fluid or electrolyte imbalanced
- significant comorbidities requiring complicated medical decision-making loops or high-intensity nursing (this is a case-by-case decision)
- complications requiring acute intervention (e.g. diabetic ketoacidosis [DKA], delirium)
- psychiatric or behavioural disturbance
- severe physical, intellectual and sensory impairment.

**For discussion with accepting consultant:** Patients whose condition will likely deteriorate, needing either high-flow oxygen or ventilation – specific attention must be paid to those already struggling on a non-rebreather mask prior to transfer (probably not for ICBF).

## Appendix 3: Risk, injury, failure, loss of kidney function and end-stage kidney disease criteria.

**TABLE 1-A3:** Risk, injury, failure, loss of kidney function and end-stage kidney disease (RIFLE) classification.

Class	GFR	UO
Risk	↑ SCr × 1.5 or ↓ GFR > 25%	< 0.5 mL/kg/h × 6 h
Injury	↑ SCr × 2 or ↓ GFR > 50%	< 0.5 mL/kg/h × 12 h
Failure	↑ SCr × 3 or ↓ GFR > 75% or if baseline SCr ≥ 353.6 μmol/L (≥ 4 mg/dL) ↑ SCr > 44.2 μmol/L(> 0.5 mg/dL)	< 0.3 mL/kg/h × 24 h or anuria × 12 h
Loss of kidney function	Complete loss of kidney function > 4 weeks	
End-stage kidney disease	Complete loss of kidney function > 3 months	

GFR, glomerular filtration rate; UO, urine output; SCr, serum creatinine.

## References

[1] Coetzee J, Taljaard JJ, Hugo SS, et al. Diabetes mellitus and COVID-19: A review and management guidance for South Africa. S Afr Med J. 2020;110(8):761–766. 32880304

[2] Mabunda S, London L, Pienaar D. An evaluation of the role of an intermediate care facility in the continuum of care in Western Cape, South Africa. Int J Health Policy Manag. 2018;7(2):167–179. 10.15171/ijhpm.2017.52 29524940 PMC5819376

[3] Williams M. First 10 patients admitted to CTICC ‘hospital of hope’ after four-week conversion [home page on the Internet]. News24. 2020 June. https://www.news24.com/news24/SouthAfrica/News/first-10-patients-admitted-to-cticc-hospital-of-hope-after-four-week-conversion-20200608

[4] Hussain A, Bhowmik B, Do Vale Moreira N. COVID-19 and diabetes: Knowledge in progress. Diabetes Res Clin Pract. 2020;162:108142. 10.1016/j.diabres.2020.108142 32278764 PMC7144611

[5] Rajpal A, Rahimi L, Ismail Beigi F. Factors leading to high morbidity and mortality of COVID-19 in patients with Type 2 diabetes. J Diabetes. 2020;12(12):895–908. 10.1111/1753-0407.13085 32671936 PMC7405270

[6] Ding Y, Wang H, Shen H, et al. The clinical pathology of severe acute respiratory syndrome (SARS): A report from China. J Pathol. 2003;200(3):282–289. 10.1002/path.1440 12845623 PMC7168017

[7] Dawei W, Bo H, Chang H, et al. Clinical characteristics of 138 hospitalized patients with 2019 novel coronavirus–infected pneumonia in Wuhan, China. JAMA. 2020;323(11):1061. 10.1001/jama.2020.1585 32031570 PMC7042881

[8] Tian S, Hu W, Niu L, Liu H, Xu H, Xiao SY. Pulmonary pathology of early-phase 2019 novel coronavirus (COVID-19) pneumonia in two patients with lung cancer. J Thorac Oncol. 2020;15(5):700–704. 10.20944/preprints202002.0220.v2 32114094 PMC7128866

[9] Zhu L, She Z, Cheng X, et al. Association of blood glucose control and outcomes in patients with COVID-19 and pre-existing type 2 diabetes. Cell Metab. 2020;31(6):1068.e3–1077.e3. 10.1016/j.cmet.2020.04.021 32369736 PMC7252168

[10] Forbes A, Murrells T, Mulnier H, Sinclair AJ. Mean Hba1c, Hba1c variability, and mortality in people with diabetes aged 70 years and older: A retrospective cohort study. Lancet Diabetes Endocrinol. 2018;6(6):476–486. 10.1016/S2213-8587(18)30048-2 29674135

[11] George CE, Mathew S, Norman G, Mukherjee D. Quality of diabetic care among patients in a tertiary care hospital in Bangalore, South India: A cross-sectional study. J Clin Diagn Res. 2015;9(7):LC07–LC10. 10.7860/JCDR/2015/11540.6215 26393148 PMC4572979

[12] Western Cape Department of Health in collaboration with the National Institute for Communicable Diseases, South Africa. Risk factors for coronavirus disease 2019 (COVID-19) death in a population cohort study from the Western Cape Province, South Africa. Clin Infect Dis. 2021;73(7)2005–2015, 10.1093/cid/ciaa1198 PMC7499501 32860699

[13] David N, Brey Z, Ismail M. Telemedicine in the Western Cape Department of Health during the first peak of the Covid 19 pandemic: Leveraging data to save lives by activating a telemedicine response. Afr J Prim Health Care Fam Med. 2021;13(1):a2954. 10.4102/phcfm.v13i1.2954 PMC8182462 34082548

[14] Johnson B, Laloraya M. A cytokine super cyclone in Covid 19 patients with risk factors: The therapeutic potential of BCG immunization. Cytokine Growth Factor Rev. 2020;54:32–42. 10.1016/j.cytogfr.2020.06.014 32747157 PMC7328575

[15] Arikan H, Ozturk S, Tokgoz B, et al. Characteristics and outcomes of acute kidney injury in hospitalized COVID-19 patients: A multicentre study by the Turkish society of nephrology. PLoS One. 2021;16(8):e0256023. 10.1371/journal.pone.0256023 34375366 PMC8354466

[16] Legrand M, Bell S, Forni L, et al. Pathophysiology of COVID-19-associated acute kidney injury. Nat Rev Nephrol. 2021;17:751–764. 10.1038/s41581-021-00452-0 34226718 PMC8256398

[17] Richardson S, Hirsch JS, Narasimhan DO. Presenting characteristics, co-morbidities and outcomes among 5700 patients hospitalised with COVID-19 in the New York City Area. JAMA. 2020;323(20):2052–2059. 10.1001/jama.2020.6775 32320003 PMC7177629

[18] Crouse AB, Grimes T, Li P, et al. Metformin use is associated with Reduced Mortality in a Diverse Population with Covid 19 and Diabetes. Front Endocrinol. 2021;11:600439. 10.3389/fendo.2020.600439 PMC7838490 33519709

[19] Gianchandani R, Esfandiari N, Ang L, et al. Managing hyperglycaemia in the COVID-19 inflammatory storm. Diabetes. 2020;69(10):2048–2053. 10.2337/dbi20-0022 32778570

[20] Huang C, Huang L, Wang Y, et al. 6 month consequences of COVID-19 in patients discharged from hospital: A cohort study. Lancet. 2021;397(10270):220–232. 10.1016/S0140-6736(20)32656-8 33428867 PMC7833295

[21] Sahoo S, Mehra A, Dua D, et al. Psychological experience of patients admitted with SARS-CoV-2 infection. Asian J Psychiatry. 2020;54:102355. 10.1016/j.ajp.2020.102355 PMC7434329 33271684

[22] Bo HX, Li W, Yang Y, et al. Post traumatic stress symptoms and attitude towards crisis mental health services among clinically stable patients with COVID-19 in China. Psychol Med. 2021;51(6):1052–1053. 10.1017/S0033291720000999 32216863 PMC7200846

[23] Saha S, Al-Rifai RH, Saha S. Diabetes prevalence and mortality in COVID-19 patients: a systematic review, meta-analysis, and meta-regression. J Diabetes Metab Disord. 2021;20(1):939–950. 10.1007/s40200-021-00779-2 33821206 PMC8012080

[24] Lopes JA, Jorge S. The RIFLE and AKIN classifications for acute kidney injury: a critical and comprehensive review. Clin Kidney J. 2013;6(1):8–14. 10.1093/ckj/sfs160. 27818745 PMC5094385

